# Electromyographic Study of Masticatory Muscle Function in Children with Down Syndrome

**DOI:** 10.3390/jcm11030506

**Published:** 2022-01-20

**Authors:** Liliana Szyszka-Sommerfeld, Magdalena Sycińska-Dziarnowska, Monika Machoy, Sławomir Wilczyński, Marzia Maglitto, Mariangela Cernera, Gianrico Spagnuolo, Krzysztof Woźniak

**Affiliations:** 1 Department of Orthodontics, Pomeranian Medical University in Szczecin, Al. Powst. Wlkp. 72, 70111 Szczecin, Poland; liliana.szyszka@gmail.com (L.S.-S.); magdadziarnowska@gmail.com (M.S.-D.); monika.machoy@pum.edu.pl (M.M.); krzysztof.wozniak@pum.edu.pl (K.W.); 2 Department of Basic Biomedical Science, Medical University of Silesia in Katowice, 3 Kasztanowa Street, 41200 Sosnowiec, Poland; swilczynski@sum.edu.pl; 3 Department of Neurosciences, Reproductive and Odontostomatological Sciences, University of Naples Federico II, 80131 Naples, Italy; mar.maglitto@gmail.com (M.M.); mariangela.cernera@icloud.com (M.C.); 4 Institute of Dentistry, I. M. Sechenov First Moscow State Medical University, 119435 Moscow, Russia

**Keywords:** asymmetry index, disability, down syndrome, masticatory muscles, muscle function, muscle hypotonia, surface electromyography

## Abstract

This study assessed the electrical activity of the masticatory muscles in both children with down syndrome (DS) and healthy children. After applying the inclusion and exclusion criteria, 30 patients aged between 7.9 and 11.8 years participated in the study. They were divided into two groups of 15: DS and non-DS. A DAB-Bluetooth device (Zebris Medical GmbH, Germany) was used to record the electromyographical (EMG) activity of the right and left temporal and of the right and left masseter muscles at rest and during maximum voluntary clenching (MVC). The asymmetry index between right and left masticatory muscle EMG activity was calculated for each position. The Mann–Whitney U test was applied to analyze the study results. There were no differences in the electrical activity of the temporal and masseter muscles at rest between the groups. During MVC, the asymmetry index for the masseter muscles was significantly higher in subjects with DS. The electrical potentials of the temporal and masseter muscles in children with DS were significantly lower compared to the corresponding parameters for healthy children when clenching.

## 1. Introduction

Down syndrome (DS) is a chromosomal disease caused by trisomy 21 that is usually accompanied by mental impairment, several comorbidities, and psychosocial limitations [1,2,3]. This genetic abnormality is characterized by variability in cognitive development and individual physical features causing unique health conditions, such as congenital heart disease, craniofacial dysmorphia, muscle hypotonia, gastrointestinal, hands-feet, renal and urogenital deformations, sleep breathing disorders including obstructive sleep apnoea, leukaemia, immune system alterations, premature dementia, Alzheimer’s disease, and others [4].

One of the most common manifestations observed in patients with DS is the generalized muscular hypotonia, especially regarding the masticatory and oropharyngeal muscles, resulting in difficulties when speaking, swallowing, and mastication [1,5]. Patients with DS may experience alterations in the stomatognathic system due to the presence of masticatory muscle dysfunction (as a result of hypotonia) and skeletal developmental abnormalities, e.g., a reduced size of the maxillary and mandibular bones and severely inhibited sagittal development in the midface region, which may result in malocclusion [1,4,6,7]. The most common dental and occlusal disorders in individuals with DS are open bite, crossbite, class III malocclusion, hypodontia, and microdontia [1,8,9]. Typical oral impairments in subjects with DS include a lack of oral motor coordination (weak jaw-closing muscles, difficulties with jaw stability, weak tongue control, and ineffective lingual lateralisation), problems during eating (poor oral seal, poor sucking, choking, belching, and food leakage from the mouth), uncontrolled facial movements, mouth breathing due to poor muscle tone, and salivary leakage [4,5,6].

Some of these abnormal muscle functions, such as those in the muscles responsible for sustaining and moving the jaw (masseter and temporal) can be accurately measured using surface electromyography (sEMG). sEMG is a non-invasive diagnostic test of muscle function that analyzes electrical potential. The visual assessment of muscular hypotonia is often subjective and is devoid of much of the relevant qualitative and quantitative information that sEMG can provide. Precise analysis of the muscle electrical activity using sEMG performed in various tests simplifies the quantitative analysis of the stomatognathic system and allows for objective muscle assessment [10,11,12,13]. The sEMG signals, conducted through tissues and recorded by surface electrodes, represent the temporal and spatial summation of the populations of nearby motor units [14]. This method is widely applied in dentistry in both clinical and research conditions [15,16]. What is important, as sEMG is simple and safe, is that it could be used in studies on children [11,17,18]. To date, information on the electromyographical (EMG) activity of the masticatory muscles in individuals with DS, particularly in the case of very young patients, is limited and the results of the existing studies are not consistent [4,17]. Therefore, further research on masticatory muscle EMG function in children with DS is needed. Early analysis of electromyographic muscle recordings in children may facilitate the development of treatment strategies to normalize muscle activity and thus contribute to the goal of achieving functional improvement in these patients. sEMG recordings could also be the basis for monitoring and evaluating the correct progress and effectiveness of therapies used in order to normalize muscle activity. Electromyographic studies of orbicularis oris muscle activity have already been conducted on DS children [19].

In light of the above, the aim of the study was to analyze the electrical activity of the anterior temporal and masseter muscles both in children with DS and in those with no DS by means of sEMG. We hypothesized that the EMG potentials of these muscles in subjects with DS at rest and during maximum clenching do not differ from those observed in non-DS subjects.

## 2. Materials and Methods

The study protocol was carried out in accordance with the guidelines of the Helsinki Declaration. The project was approved by the Local Bioethics Committee of the Pomeranian Medical University (project number KB-0012/08/15). After obtaining comprehensive information about the goals and methods used in the study, written informed parental consent was obtained, as well as the children’s assent for voluntary participation in the investigations and procedures performed.

Initially, 70 children, aged between 7.9 and 11.8 years and of both genders, were invited to participate in the study. They were recruited from among children with mixed dentition who had been referred for orthodontic treatment to the Developmental Facial Abnormalities Clinic and Orthodontics Outpatient Clinic in Szczecin, Poland between August and October 2019. Among them there were 30 subjects with DS and 40 individuals without DS. The application of the adopted inclusion and exclusion criteria resulted in 40 of the subjects being excluded from the study and 30 of the participants qualifying for the next stages of the research. They were divided into two groups of 15 individuals each: DS (mean age 10.1 ± 1.1) and non-DS (mean age 9.8 ± 1.0).

The inclusion criteria for the study groups were as follows: both genders, mixed dentition, Angle Class I occlusion, ability to understand and respond to verbal commands, e.g., “open or close the mouth” and “clench as hard as possible”, and voluntary consent to participate in the study. The control group (non-DS) included children with normal functional occlusion (a good relationship between the dental arches).

The exclusion criteria for the study groups were as follows: systemic diseases affecting the muscles, neuromuscular illness or disease affecting neuromuscular performance, medications that could affect muscle function, history of trauma or surgical treatment in the orofacial area, oral parafunctional habits, including bruxism, and class II and class III malocclusions.

First, anamnesis of a patient was performed in order to investigate oral and general health, treatments, the taking of medication, medical and family history, parafunctional habits, and psychological aspects. A follow-up extra- and intraoral clinical examination was then performed. This provided information about the subjects’ facial profile and details regarding their masticatory organ, e.g., the type of lip seal, mode of breathing, swallowing pattern, as well as the presence of temporomandibular disorder or bruxism. The intraoral analysis of the dental arches on three planes was used to investigate the occlusal features, including vertical overlap, overjet, angle class, crossbite, and open bite.

The next part of the children’ examination involved an evaluation of masticatory muscle function so as to obtain the surface electromyography records.

A DAB-Bluetooth electromyography system was used (Zebris Medical GmbH, Isny im Allgäu, Germany). This device was characterized by amplifier gains of 1000 times, a high-pass filter of 7 Hz–5 kHz, a channel sampling rate of 1 kHz, a 12-bit dynamic resolution range, and an input impedance for analogue channels of 146 kΩ. Disposable Ag/AgCl bipolar surface electrodes (Noraxon Dual Electrode, Noraxon, Scottsdale, AZ, USA) with a constant distance of 20 mm between the electrodes were bilaterally positioned on the right and left anterior temporal (RTA and LTA) as well as on the right and left masseter (RMM and LMM) muscles parallel to the direction of the fibers. During the sEMG recordings, the subjects were comfortably seated in an upright position in a dentist’s chair with the head in a natural position [20]. The placement of the surface electrodes followed the technique described by Ferrario et al. [21]: the anterior portion of the temporal muscle—vertically along the anterior margin of the muscle and approximately over the coronal suture; the superficial part of the masseter muscle—parallel to the muscular fibers with the upper pole of the electrode attached at the intersection between the tragus-labial commissura and the exocanthion-gonion lines. The reference electrode was placed lower and behind the right ear.

Before taking the sEMG recordings the patient’s skin was cleaned with a 70% ethyl alcohol solution to decrease impedance. After attaching the surface electrodes, a Metex P-10 measuring device (Metex Instruments Corporation, Seocho-gu, Seoul, Korea) was used to test the impedance and confirm that the investigated region had been properly prepared. Prior to the recordings, skin impedance had to be approximately 1 × 10^3^–30 × 10^3^ Ω.

Prior to the tests, the participants received a brief training session to familiarize them with the tasks. The sEMG examinations of the temporal and masseter muscles were then performed in accordance with the following protocol:

(1) in mandibular rest position;
(2) during maximum voluntary clenching (MVC) (keeping teeth clenched as strongly as possible in the intercuspal position for a duration of 5 s);
(3) during maximum voluntary clenching with two cotton rolls bilaterally positioned on the occlusal surfaces of posterior teeth (clenching as strongly possible for a duration of 5 s).

A rest period of at least 5 min was allowed between each recording. All the tasks had to be repeated at least three times and the average of the last two sEMG recordings was written down [22,23].

The differential raw sEMG signals were filtered, amplified, and digitized. Then, the EMG data were recorded by the computer and normalized to ensure further reliable analysis. The clenching test with two cotton rolls (standardization test) provides reference EMG values for the subsequent normalization. For each of the analyzed muscles, the mean EMG potentials during MVC on cotton rolls were set to 100% and all EMG values obtained during MVC and at rest were divided by the mean EMG data of the normalization record on the cotton rolls. Finally, the EMG potentials were expressed as a percentage of the MVC value using cotton rolls (μV/μV%). The normalization process allows for the elimination of any anatomical and technical variability due to skin and electrode impedance, electrode placement, and relative muscle hypo- or hypertrophy [24,25,26,27,28].

Asymmetry between the left (L) and right (R) masticatory muscle activity was determined quantitatively using the asymmetry index (As, unit %, range from 0% to 100%) according to the following equation [29]:

As = ∑i = 1N|Ri − Li|/∑i = 1N(Ri + Li) × 100

The reproducibility of the recording protocol was tested by means of duplicate sEMG recordings of 10 children made by the same operator. Between the two sEMG measurements the children were asked to relax for a duration of 15 min.

For the purposes of the statistical analysis STATISTICA 13.0 PL for Windows software package (StatSoft Poland, Cracow, Poland) was used. Normality was assessed with the Shapiro–Wilk test. The data were not distributed normally and the non-parametric Mann–Whitney U test was applied to compare the EMG results between the DS and control groups. A value of *p* < 0.05 was considered significant.

## 3. Results

Table 1 shows the characteristics of the study participants. Subjects with DS were characterized by abnormal lip seal (100%), atypical swallowing (66.7%), mouth breathing (53.3%), angle class I (100%), and malocclusions, such as posterior crossbites (66.7%), anterior crossbites and anterior open bites (33.3%), and lateral open bites (26.7%). Most of the DS individuals had a straight facial profile (46.7%). All the subjects with no DS had normal lip seal, nose breathing, normal swallowing pattern, and angle class I occlusion with a good relationship between the dental arches (no malocclusion). Most of the non-DS subjects had a straight facial profile (73.3%).

There were no significant differences (*p* > 0.05) between independent duplicated sEMG recordings in all analyzed variables.

The EMG activity of the temporal and masseter muscles at rest was similar in both the children with DS and in the healthy control subjects (*p* > 0.05) (Table 2).

The statistical analysis revealed significant differences between the DS and non-DS groups in the electrical activity of the masticatory muscles during MVC. The EMG potentials of the temporal and masseter muscles were significantly lower in the DS children compared to the subjects with no diagnosis of DS (for the RTA *p* = 0.023; for the LTA *p* = 0.005; TA_mean_ *p* = 0.009; for the RMM *p* = 0.010; for the LMM *p* < 0.001; MM_mean_ *p* = 0.001) (Table 3).

No statistically significant intergroup differences were observed in the asymmetry index for the right and left temporal and masseter muscles at rest or for the temporal muscles during clenching (*p* > 0.05). The asymmetry index for the masseter muscles during MVC was significantly higher in the subjects with DS than in children in the control group (*p* < 0.001) (Table 4).

## 4. Discussion

This study provided an electromyographical analysis of masticatory muscle function in subjects diagnosed with a particular congenital anomaly, namely down syndrome. Children with DS had altered masticatory muscle activity compared to controls. The results of the study showed significantly lower electrical potentials of the temporal and masseter muscles during MVC in children with DS compared with the non-DS individuals.

The data obtained in the present research can be compared with the findings of Nęcka et al. [17], who analyzed the electrical potentials of the temporal and masseter muscles by means of electromyography in children with DS and healthy subjects with marked hypotonia of the facial muscles at rest when the lips are placed in a whistling position and during maximum intercuspation. In contrast to our study, they observed no significant differences in the EMG signals between analyzed groups during these situations in the case of either the temporal or masseter muscles. It can be speculated that these different results may be due to differences in the selection criteria of the study group. Since many variables may be associated with the electromyographical pattern of the masticatory muscles, our study comprised only individuals with class I occlusions, thereby ensuring homogeneity of the sample and reducing the number of interfering factors. It should also be noted that our control group included children without malocclusions, with normal lip seal, nasal breathing and normal swallowing pattern, whereas the DS group included children with malocclusions, such as crossbite and open bite, as well as subjects with lip incompetence, mouth breathing and atypical swallowing. The study by Nęcka et al. [17] did not discuss the characteristics of the study population and did not precisely define the inclusion and exclusion criteria for the study groups.

An analysis of masticatory muscle function in patients with Down syndrome was also the subject of a study conducted by Gomes et al. [4]. The authors assessed the electrical activity of the masseter and temporal muscles at rest and during MVC in adult patients with DS aged between 19 and 40 years. Similarly to our study, they found that the EMG potentials of the right and left temporal muscles and of the right and left masseter muscles during MVC were significantly lower in the DS group than in the control group, and this indicated hypotonia in both muscles. Furthermore, they observed that the EMG activity of the RMM and LMM muscles in the control group and on the unilateral side was significantly greater when compared with their respective positioner muscles. However, the mean values of the EMG activity of the masseter and temporal muscles in the DS group were bilaterally similar. The authors found no significant differences in the EMG potentials at rest between the groups.

In our study, the electromyographic activity of the temporal and masseter muscles at rest was also similar in both children with DS and in healthy children. The minimal EMG activity of the masticatory muscles in both groups may indicate harmony between the agonist and antagonist muscles. This situation allowed for the preservation of the interocclusal clearance or “freeway space”, which in turn helps to maintain the physiological resting position of the mandible [4].

The asymmetry index of the masseter muscles during MVC was significantly higher in patients with DS than in the controls. This indicates differential left–right muscle activity in individuals with DS and could be a consequence of unbalanced occlusion, bilateral intercuspal interferences and a preferred chewing side caused by neuromuscular action and/or craniofacial alterations. Moreover, asymmetric masticatory function in children with DS is important in the context of its consequences, such as asymmetric facial growth [30].

Our experiment reveals that the EMG potentials of the temporal and masseter muscles in the DS group were much lower when clenching and this may indicate masticatory muscle hypofunction in DS children through sEMG recordings. Although the pathogenesis of muscular hypotonia in people with DS is not fully understood, previous studies have shown that structural and functional changes in the central nervous system are the main cause of muscle weakness [4,31]. When interpreting these study results, it should also be borne in mind that many variables may be associated with the electromyographical pattern of the masticatory muscles, e.g., malocclusion, type of lip seal, mode of breathing, or pattern of swallowing. In our study, children with DS had various malocclusions. The most common malocclusion in the DS group was posterior crossbite, both unilateral and bilateral. This may be one of the factors possibly associated with masticatory muscle function in Down syndrome patients. A link between changes in the EMG activity of the masticatory muscles and the presence of posterior crossbite has been confirmed in many previous studies [11,18,32]. It is known that the number of posterior occlusal contacts has an impact on the masticatory muscle activity when maximum effort is exerted [33]. A larger number of posterior contacts provide stable intercuspal support. This allows the elevator muscles to achieve increased muscular activity during clenching or chewing. The reduced number of occlusal contacts in individuals with DS and posterior crossbite may have limited stimulation from periodontal receptors, which may result in reduced muscle activity. Furthermore, to avoid occlusal changes, this hypofunction of the masticatory muscles during clenching may also be considered an effective protective mechanism in the masticatory organ [11,34,35].

It should also be noted that most of the children with DS were diagnosed with atypical swallowing and mouth breathing, and all had lip incompetence. A number of studies have indicated an association between these variables and masticatory muscle activity [36,37,38]. Ikenaga et al. [36] showed that the electrical potentials of the masticatory muscles while chewing food for mouth breathing were significantly lower than the EMG values for nose breathing. They demonstrated that mouth breathing decreases chewing activity and reduces the vertical effect on the posterior teeth. Störmer and Pancherz [37] observed differences between subjects with open bite and atypical swallowing and patients with a typical swallowing pattern when it came to the electromyographical activity of the temporal and masseter muscles during saliva and water swallowing when chewing peanuts and in conditions of maximal biting in the intercuspal position. Similarly, Lipari et al. [38] pointed to the existence of changes in the EMG signals of the temporal muscles in children with incompetent lips. They observed that EMG activity at rest, when speaking, and when swallowing was lower in patients with lip incompetence than in subjects with normal lip seal.

Our study has a number of limitations. Firstly, the research sample was represented by only a small study population. Moreover, the participants in the study groups present different characteristics, including occlusal characteristics. Since DS subjects had some malocclusions and this is one of the factors that may result in changes in muscle electrical activity, the results of the study should be interpreted with some reservations. Therefore, further research should be conducted on a larger and more homogeneous group of patients so as to support and confirm the study results and to identify the possible variables that could be associated with masticatory muscle EMG activity in DS individuals.

As mentioned earlier, sEMG is a non-invasive technique for evaluating muscle function and efficiency by recording muscle activity from the surface over the muscle on the skin using a pair of electrodes. This method allows for objective muscle assessment and can provide some relevant qualitative and quantitative information [16]. In this way, it may be a useful diagnostic tool in children with muscle hypotonia. However, it should be noted that a precise diagnosis of muscle hypotonia requires a medical history, clinical examination, and other additional diagnostic tests, including electrophysiological, neuroimaging, laboratory and genetic tests or muscle and nerve biopsies [39,40]. In addition, surface electrodes were used in this study to evaluate the EMG potentials of the masticatory muscles. The sEMG method has some drawbacks, such as its sensitivity to impedance imbalance, which may reduce the accuracy of sEMG recordings, as well as the tendency to identify overlapping EMG potentials from multiple muscle fibers, which limits the ability of sEMG to assess overall muscle activity. However, the fixed distance between electrodes, a standard electrode positioning procedure, and the analysis of sEMG data based on the normalization procedure reduce these problems.

It should also be noted that the electrical activity of the masticatory muscles was recorded during the most important tests from a biomechanical point of view, such as rest and maximum voluntary contraction (MVC). MVC represents the maximum voluntary isometric activation of a muscle and provides a physiological reference point. It is a highly reproducible activity. Normalization of the corresponding EMG signals is usually based on measurements during MVC. The main assumption of the normalization procedure is the constancy and good reproducibility of the forces generated during maximum voluntary clench [13,41,42]. From a clinical point of view, it is important that an alteration of the pattern of masticatory muscle electrical activity during MVC in the intercuspal position can affect muscle fatigue, and can, as a consequence, have an impact on every function they perform in the stomatognathic system. In light of the above, the analysis of the masticatory muscle EMG activity during MVC is in direct correlation with the function of the masticatory system [43]. On the other hand, among the dynamic activities, mastication is one of the most frequently analyzed by means of sEMG. However, due to the high variability of the movements that contribute to this activity, the evaluation of mastication is very difficult [13,44]. For these reasons, and since our study included a small number of participants, we did not analyze the masticatory muscle activity during mastication in this project.

## 5. Conclusions

The sEMG recordings showed that in comparison to non-DS individuals, children with DS have lower EMG potentials of the temporal and masseter muscles during clenching. Further research should be conducted on larger groups of subjects so as to confirm the study results and to identify the possible variables that could be associated with masticatory muscle EMG activity in DS individuals.

## Figures and Tables

**Table 1:** The characteristics of the study participants.

Variable		DS Group Mean Age 10.1 ± 1.1	Non-DS Group Mean Age 9.8 ± 1.0
		*n (*%)	*n (*%)
Gender	Females	6 (40.0)	7 (46.7)
	Males	9 (60.0)	8 (53.3)
Facial profile	Straight	7 (46.7)	11 (73.3)
	Concave	3 (20.0)	4 (26.7)
	Convex	5 (33.3)	0
Lip seal	Normal	0	15 (100)
	Abnormal	15 (100)	0
Mouth breathing	No	7 (46.7)	15 (100)
	Yes	8 (53.3)	0
Atypical swallowing	No	5 (33.3)	15 (100)
	Yes	10 (66.7)	0
Vertical overlap	≥0 < 3 mm	6 (40.0)	15 (100)
	≥3 mm	4 (26.7)	0
	Reverse	5 (33.3)	0
Overjet	≥0 < 3 mm	5 (33.3)	15 (100)
	≥3 mm	5 (33.3)	0
	Negative	5 (33.4)	0
Angle Class	I	15 (100)	15 (100)
	II	0	0
	III	0	0
Posterior crossbite	No	5 (33.3)	15 (100)
	Yes	10 (66.7)	0
Lateral open bite	No	11 (73.3)	15 (100)
	Yes	4 (26.7)	0

DS: Down syndrome.

**Table 2:** EMG activity of the masticatory muscles at rest in the DS and non-DS groups.

EMG Activity (µV/µV%)	DS Group						Non-DS Group						
	*n*	Min	Q1	Mdn	Q3	Max	*n*	Min	Q1	Mdn	Q3	Max	*p*
RTA	15	3.6	5.1	7.6	8.0	9.2	15	3.4	4.1	5.4	7.3	9.9	0.329
LTA	15	4.6	6.0	6.9	7.8	8.0	15	2.9	4.6	5.3	9.1	13.0	0.110
TA_mean_	15	4.1	5.6	7.0	7.9	8.1	15	3.1	4.3	5.0	8.5	11.4	0.263
RMM	15	4.8	6.2	7.2	9.4	11.9	15	4.3	5.3	7.5	9.1	11.6	0.648
LMM	15	4.7	5.4	7.9	8.9	10.7	15	5.1	5.8	7.3	7.8	11.9	0.245
MM_mean_	15	4.9	6.3	7.9	9.5	10.4	15	4.7	6.2	7.4	8.1	11.7	0.407

EMG: electromyographic, DS: Down syndrome, RTA: right anterior temporal muscle, LTA: left anterior temporal muscle, TA: anterior temporal muscles, RMM: right masseter muscle, LMM: left masseter muscle, MM: masseter muscles, Min: minimum, Q1: first quartile, Mdn: median, Q3: third quartile, Max: maximum. Significance was measured using the Mann–Whitney U test.

**Table 3:** EMG activity of the masticatory muscles during MVC in the DS and non-DS groups.

EMG Activity (µV/µV%)	DS Group						Non-DS Group						
	*n*	Min	Q1	Mdn	Q3	Max	*n*	Min	Q1	Mdn	Q3	Max	*p*
RTA	15	38.4	65.8	75.8	89.8	117.1	15	67.7	78.3	97.8	113.7	116.8	0.023
LTA	15	32.1	53.2	71.2	82.1	120.5	15	56.9	89.8	113.3	117.4	125.6	0.005
TA_mean_	15	43.9	57.3	72.9	80.5	118.8	15	65.1	78.7	106.0	115.3	119.6	0.009
RMM	15	35.5	61.1	73.4	116.2	126.5	15	80.5	83.2	111.5	126.2	131.8	0.010
LMM	15	39.8	55.7	72.6	92.5	112.6	15	71.3	90.8	110.8	119.8	123.5	<0.001
MM_mean_	15	41.0	64.5	71.1	109.5	119.1	15	75.9	87.0	113.8	121.8	125.5	0.001

MVC: maximum voluntary clenching, EMG: electromyographic, DS: Down syndrome, RTA: right anterior temporal muscle, LTA: left anterior temporal muscle, TA: anterior temporal muscles, RMM: right masseter muscle, LMM: left masseter muscle, MM: masseter muscles, Min: minimum, Q1: first quartile, Mdn: median, Q3: third quartile, Max: maximum. Significance was measured using the Mann–Whitney U test.

**Table 4:** Asymmetry index of the masticatory muscles in the DS and non-DS groups.

As (%)	DS Group						Non-DS Group						
	*n*	Min	Q1	Mdn	Q3	Max	*n*	Min	Q1	Mdn	Q3	Max	*p*
**Rest**													
TA	15	0.6	3.2	7.4	12.2	21.7	15	1.1	4.9	6.2	10.2	14.1	0.836
MM	15	1.6	6.9	12.6	14.5	20.3	15	1.3	3.7	8.5	14.5	21.2	0.481
**MVC**													
TA	15	1.4	4.2	11.0	18.7	27.0	15	0.3	1.9	5.0	11.1	15.8	0.089
MM	15	3.2	6.9	12.9	3.7	24.1	15	1.3	3.1	3.8	6.0	14.8	<0.001

DS: Down syndrome, TA: anterior temporal muscles, MM: masseter muscles, Min: minimum, Q1: first quartile, Mdn: median, Q3: third quartile, Max: maximum. Significance was measured using the Mann–Whitney U test.

## Data Availability

The datasets used to support the conclusions of this article are included within the article. Access to other data will be considered by the corresponding author upon request.
